# Use of Stingless Bee Propolis and Geopropolis against Cancer—A Literature Review of Preclinical Studies

**DOI:** 10.3390/ph14111161

**Published:** 2021-11-14

**Authors:** Francisco Assis Nascimento Pereira, Josianne Rocha Barboza, Cleydlenne Costa Vasconcelos, Alberto Jorge Oliveira Lopes, Maria Nilce de Sousa Ribeiro

**Affiliations:** Laboratório de Farmacognosia, Departamento de Farmácia, Campus Bacanga, Universidade Federal do Maranhão, Av. dos Portugueses, 1966, São Luís 65080-805, Maranhão, Brazil; josi.anne.r@hotmail.com (J.R.B.); cleydlenne@yahoo.com.br (C.C.V.)

**Keywords:** stingless bee products, new anticancer agents, propolis, geopropolis

## Abstract

Cancer is one of the major maladies affecting humankind and remains one of the leading causes of death worldwide. The investigation of the biological activities of stingless bee products, especially propolis and geopropolis, has revealed promising therapeutic properties, especially in the research on new antineoplastic agents. This literature review of preclinical trials, involving biological assays of antitumor activity and identification of the chemical composition of propolis and geopropolis of stingless bee species, describes the cytotoxicity in tumor lineages (breast, lung, ovarian, liver, mouth, pharynx, larynx, colon, stomach, colorectal, cervix, kidney, prostate, melanoma, human glioblastoma, canine osteosarcoma, erythroleukemia, human chronic myelocytic leukemia, and human promyelocytic leukemia) of propolis and geopropolis of 33 species of stingless bees. The chemical composition of propolis and geopropolis was identified, indicating that these belong to the chemical classes of phenolic acids, flavonoids, coumarins, benzophenones, anthraquinones, alkaloids, terpenes, steroids, saponins, fatty acids, and carbohydrates and are possibly responsible for the cytotoxicity in tumor cells. Apoptosis was one of the main mechanisms of cytotoxicity of extracts and substances isolated from stingless bee products. Although the results found are encouraging, other preclinical studies and clinical trials are essential for the discovery of new anticancer agents.

## 1. Introduction

Stingless bees, also known as meliponines, live in colonies and are characterized by having atrophied stingers [1]. They are social insects of great diversity and wide geographic distribution, occupying almost all of Latin America and Africa, besides southeast Asia and northern Australia [2,3,4]. There are more than 600 described species, and they are spread across all tropical and subtropical areas of the globe [5]. Among the genera with the largest number of known species are *Plebeia*, *Trigona*, *Melipona*, *Scaptotrigona*, and *Trigonisca* [6].

Meliponines make great contribution to environmental conservation, as they perform pollination of native plant species and contribute to a reduction in deforestation and environmental damage [4,7]. In addition, they are commercially known for their role in the production of natural products, such as honey, wax, royal jelly, propolis, and geopropolis and accumulation of pollen [3,4,8,9].

Propolis is a mixture of salivary secretions and plant resins collected by bees and is produced to seal the hive and prevent the entry of air and invading insects, besides having antimicrobial activity, protecting the colony from diseases [10,11].

Some meliponin species mix propolis with clay or soil. The result of this mixture is a resinous material more rigid than propolis. Despite the differences in the formation of both products, geopropolis has similar functions to propolis regarding the protection of the hive [12].

Different biological activities of propolis and geopropolis have been investigated worldwide, including antioxidant [13,14,15,16], antimicrobial [15,17], antileishmanial [18], antiviral [19], anti-inflammatory [20], healing [21], and antitumor [8,22,23,24,25] action.

The evaluation of the antitumor activity of propolis and geopropolis has been the object of research in several study groups. These stingless bee products have already been tested in many tumor models of head and neck, lung, liver, pancreas, kidney, prostate, skin, breast, gastric, and colon cancer, the results of which suggest the potential use of these natural compounds as part of complementary medical treatment of human tumors [17,24,26,27].

Considering the importance of natural products for the development of antineoplastic drugs, the present study conducted a literature review of studies of the effect of propolis and geopropolis produced by species of stingless bees against different tumor lineages and the identification of their chemical compounds responsible for the biological activity.

## 2. Results and Discussion

The selection of articles by primary search identified 2080 articles, of which 1622 were in ScienceDirect, 126 were in PubMed, 310 were in Scopus, and 22 were in Scielo. Articles indexed in two or more databases were considered only once. After the initial screening of titles, abstracts, and keywords, 31 articles were selected, as the others did not meet the inclusion criteria.

A total of 33 species of stingless bees producing propolis and geopropolis with antitumor potential were identified, of which 20 species (*Scaptotrigona affinis postica*, *Scaptotrigona bipunctata*, *Scaptotrigona depilis*, *Scaptotrigona* sp., *Melipona quadrifasciata quadrifasciata*, *Melipona quadrifasciata anthidioides*, *Melipona orbignyi*, *Trigona* spp., *Trigona incisa*, *Trigona apicalis*, *Trigona fuscobalteata*, *Trigona fuscibisca*, *Trigona laeviceps*, *Trigona sirindhornae*, *Tetragonisca fiebrigi*, *Tetrigona apicalis*, *Tetragonula pagdeni*, *Tetragonula biroi*, *Heterotrigona itama*, *Heterotrigona bakeri*, *Homotrigona fimbriata*, *Tetragonula testaceitarsis*, *Tetragonula sarawakensis*, *Tetragonula fuscobalteata*, *Tetragonula laeviceps*, *Lepidotrigona terminata*, *Lepidotrigona ventralis*, *Geniotrigona thoracica*, *Lisotrigona furva*, and *Plebeia remota*) are producers of propolis and three species (*Melipona fasciculata*, *Melipona mondury*, and *Melipona scutellaris*) are producers of geopropolis (Table 1).

Only two studies on the propolis cytotoxicity of stingless bees (*Scaptotrigona aff. postica* and *Tetragonula biroi*) were found in animal models [28,29] with the remaining studies being in vitro tests. These stingless bee products have already been tested in vitro on tumor cell lines, such as breast (MDA-MB-231, MCF-7, and BT-474), lung (A549, H460, SK-LU-1, and ChaGo-1), ovarian (ES2, A2780, NCI-ADR/RES, and OVCAR-03) cancer, liver (HepG2), mouth (KB), pharynx (HN30 and HN31), larynx (HEp-2), colon (CaCo-2, COLO205, SW620, and KM12), stomach (KATO-III, AGS, MKN-45, NUGC-4, and MKN-74), colorectal (HRT-18), cervix (HeLa), kidney (786-0), prostate (PC-3), melanoma (UACC-62, SK-MEL-28, and B16-F10), human glioblastoma (U251 and U343), canine osteosarcoma (OSA), and leukemia (K562 and HL-60) (Table 1).

**Figure 1:**
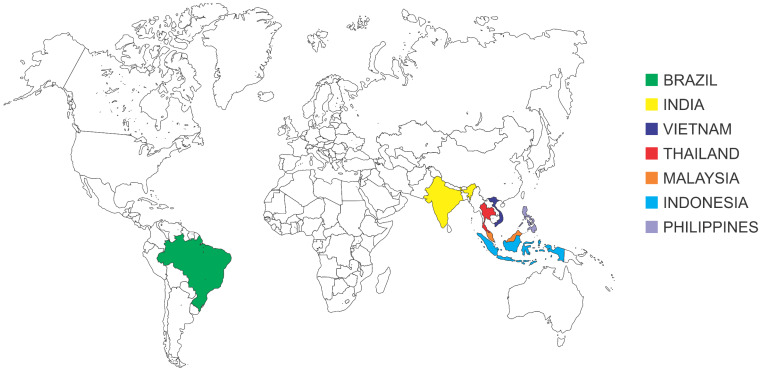
Locations of stingless bee species producing propolis and geopropolis with antitumor potential worldwide.

Stingless bee products with antitumor potential come from seven countries (Brazil, India, Indonesia, Thailand, Malaysia, Philippine, and Vietnam) (Figure 1).

**Table 1:** Propolis and geopropolis extracts of stingless bee species with anticancer activity in tumor cell lines.

Bee Species	Place of Origin	Product	Type of Preparation	Tumor Cells	Result	Type of Test	Chemical Identification	Ref.
*Melipona fasciculata* (Smith 1854)	Maranhão, Brazil	Geopropolis	Hydroethanolic extract	Canine osteosarcoma (OSA)	Dose- and time-dependent cytotoxicity	In vitro	No	[23]
				Human epidermoid laryngeal carcinoma (HEp-2)	Decrease in cell viability from 25 to 100 μg/mL		Yes ^a^	[30]
				Human epidermoid laryngeal carcinoma (HEp-2)	Inhibition of cell proliferation and migration		No	[25]
				Lung cancer (A549 and H460) and ovarian cancer (ES2 and A2780)	Dose- and time-dependent cytotoxicity		Yes ^a^	[8]
*Melipona scutellaris* (Latreille 1811)	Bahia, Brazil		Ethanolic extract	Glioma (U251), melanoma (UACC-62), breast (MCF-7), multidrug-resistant ovarian (NCI-ADR/RES), kidney (786-0), lung (NCI-H460), prostate (PC-3), and ovary (OVCAR-03)	Anti-proliferative activity		Yes ^a^	[24]
*Melipona mondury* (Smith 1863)	Bahia, Brazil		Hydroethanolic extract	B16-F10 (melanoma murine), HepG2 (human hepatocellular carcinoma), K562 (human chronic myeloid leukemia), and HL-60 (human promyelocytic leukemia)	IC_50_ 24.2 to 46.6 μg/mL		Partially	[15]
*Melipona quadrifasciata quadrifasciata* (Lepeletier 1836)	Paraná, Brazil	Propolis	Ethanolic extract	MDA-MB-231 (triple-negative human breast adenocarcinoma), MCF-7 (human breast adenocarcinoma), HeLa (human cervical adenocarcinoma), HepG2 (human hepatocellular carcinoma), HRT-18 (human colorectal adenocarcinoma)	IC_50_ 97.53 to 155.1 μg/mL	In vitro	Yes ^a^	[17]
*Melipona quadrifasciata anthidioides* (Lepeletier 1836)	Mato Grosso do Sul, Brazil	Propolis	Ethanolic extract	Erythroleukemia cell line (K562)	Decrease in cell growth to 21.2% ± 4.1% at 500 µg/mL	In vitro	Yes ^a^	[31]
	Santa Catarina, Brazil		Ethanolic extract	Human melanoma (SK-MEL-28)	Decreased migration and invasion of melanoma cells		Yes ^a^	[32]
*Melipona orbignyi* (Guérin-Méneville 1844)	Mato Grosso do Sul, Brazil		Ethanolic extract	Erythroleukemia cell line (K562)	Decrease in cell viability to less than 25% at 500 µg/mL		Yes ^b^	[33]
*Trigona* spp.	Maharashtra, India		Hydroethanolic extract	Human breast adenocarcinoma (MCF-7), human colon adenocarcinoma (HT-29), human epithelial colorectal adenocarcinoma (CaCo-2), and murine melanoma cell lines (B16F1).	Time- and dose-dependent cytotoxicity IC_50_ 250 µg/mL		No	[26]
	Indonesia		Hydroethanolic extract	Breast (MCF-7)	Decrease in cell growth to 47.71%		Partially	[34,35]
*Trigona sirindhornae* (Michener and Boongird 2004)	Chantaburi, Thailand		Dichloromethane extract	Primary lesions of the pharynx (HN30) and lymph node metastases (HN31)	Dose-dependent cytotoxicity		No	[36]
*Tetragonula pagdeni* (Schwarz 1939)	Chanthaburi, Thailand	Propolis	Methanolic extract	Squamous cell carcinoma of the mouth (KB), hepatocellular carcinoma (HepG2), colon adenocarcinoma (CaCo-2), and melanoma (SK-MEL-28)	Cytotoxicity IC_50_ 33.38 to 80.81 μg/mL	In vitro	Yes ^b^	[27]
*Tetragonula testaceitarsis* (Cameron 1901)	Kalimantan, Indonesia		Ethanolic extract	Human breast cancer (MCF-7), human cervical adenocarcinoma (HeLa), and human colon cancer (CaCo-2)	Moderate decrease in cell viability to 75 μg/mL		No	[37]
*Tetragonula sarawakensis* (Schwarz 1939)							No	
*Tetragonula fuscobalteata* (Cameron 1908)							No	
*Tetragonula laeviceps* (Smith 1857)							No	
*Tetragonisca fiebrigi* (Schwarz 1938)	Mato Grosso do Sul, Brazil	Propolis	Ethanolic extract	Erythroleukemia cell line (K562)	Dose-dependent cytotoxicity		Yes ^a^	[20]
*Trigona incisa* (Sakagami and Inoue 1989)	Kalimantan, Indonesia	Propolis	Methanolic extract	Colon (SW620), liver (HepG2), stomach (KATO-III), lung (ChaGo-1), and breast (BT-474)	Anti-proliferative activity	In vitro	Yes ^b^	[38,39,40]
*Trigona apicalis* (Smith 1857)							No	[38]
*Trigona fuscobalteata* (Cameron 1908)							No	
*Trigona fuscibisca* (Friese 1900)								
*Heterotrigona itama* (Cockerell 1918)			Ethanolic extract	Human breast cancer (MCF-7), human cervical adenocarcinoma (HeLa), and human colon cancer (CaCo-2)	Moderate decrease in cell viability to 75 μg/mL	In vitro	No	[37]
*Heterotrigona bakeri* (Cockerell 1919)							No	
*Homotrigona fimbriata* (Smith 1857)							Yes ^b^	
*Lepidotrigona terminata* (Smith 1878)	Chanthaburi, Thailand	Propolis	Methanolic extract	Squamous cell carcinoma of the mouth (KB), hepatocellular carcinoma (HepG2), colon adenocarcinoma (CaCo-2), and melanoma (SK-MEL-28)	Cytotoxicity IC_50_ 74.30 to 264.78 μg/mL	In vitro	No	[27]
*Trigona laeviceps* (Smith 1857)	Samut Songkram, Thailand		Aqueous extract	Colon (SW620)	Decrease of cell viability to 23%		No	[41]
			Ethanolic extract	Colon (SW620), breast (BT-474), liver (HepG2), lung (ChaGo), and stomach (KATO-III)	Anti-proliferative activity IC_50_ 19.9 to 36.19 μg/mL		No	[42]
*Lepidotrigona ventralis* (Smith 1857)	Chanthaburi, Thailand		Methanolic extract	Squamous cell carcinoma of the mouth (KB), hepatocellular carcinoma (HepG2), colon adenocarcinoma (CaCo-2), and melanoma (SK-MEL-28).	Cytotoxicity IC_50_ 96.58 to 565.19 μg/mL		No	[27]
*Geniotrigona thoracica* (Smith 1857)	Perak, Malaysia		Ethanolic extract	Human breast adenocarcinoma (MCF-7)	Growth inhibition IC_50_ 38.9 μg/mL		No	[43]
*Plebeia remota* (Holmberg 1903)	Paraná, Brazil		Ethanolic extract	MDA-MB-231 (triple-negative human breast adenocarcinoma), MCF-7 (human breast adenocarcinoma), HeLa (human cervical adenocarcinoma), HepG2 (human hepatocellular carcinoma), and HRT-18 (human colorectal adenocarcinoma)	IC_50_ 41.76 to 76.1 μg/mL		Yes ^a^	[17]
*Tetragonula biroi* (Friese 1898)	Lagunas, Philippines		Ethanolic extract	Gastric cancer cell lines (AGS, MKN-45, NUGC-4, and MKN-74)	Regression of macroscopic and histological lesions	In vitro and in vivo	Yes ^a^	[29]
*Scaptotrigona aff. postica* (Latreille 1807)	Maranhão, Brazil	Propolis	Hydroethanolic extract	Ehrlich solid tumor	Inhibition of tumor progression	In vivo	Partially ^a^	[28]
*Scaptotrigona bipunctata* (Lepeletier 1836)	Paraná, Brazil		Ethanolic extract	MDA-MB-231 (triple-negative human breast adenocarcinoma), MCF-7 (human breast adenocarcinoma), HeLa (human cervical adenocarcinoma), HepG2 (human hepatocellular carcinoma), and HRT-18 (human colorectal adenocarcinoma)	Cytotoxicity IC_50_ 54.89 to 112.23 μg/mL	In vitro	Yes ^a^	[17]
*Scaptotrigona bipunctata* (Lepeletier 1836)	Santa Catarina, Brazil		Ethanolic extract	Human melanoma (SK-MEL-28)	Decreased migration and invasion of melanoma cells		Yes ^a^	[32]
*Scaptotrigona depilis* (Moure 1942)	Mato Grosso do Sul, Brazil		Ethanolic extract	Human erythroleukemia cell line (K562)	Decrease in cell growth to 32.6 ± 3.2% at 500 μg/mL		Yes ^a^	[31]
*Scaptotrigona* sp.	Maranhão, Brazil		Ethanolic extract	Human glioblastoma (U251 and U343)	Anti-proliferative activity		No	[22]
*Tetrigona apicalis* (Smith 1857)	Perak, Malaysia		Ethanolic extract	Human breast adenocarcinoma (MCF-7)	Proliferation inhibition IC_50_ 32.70 μg/mL		Yes ^a^	[44]

a = chemical composition detailed in Table 2. b = compound isolation shown in Figure 2.

### 2.1. Cytotoxicity Tests

#### 2.1.1. Ehrlich Tumor

Ehrlich tumor is a model of mammary adenocarcinoma of female mice that is used in the evaluation of antitumor drugs [45]. According to Araújo et al. [28], animals inoculated by Ehrlich tumor cells in the paws treated with the hydroethanolic propolis extract produced by *Scaptotrigona aff*. *postica* at doses of 0.5 and 5 mg/kg showed significant inhibition in tumor development from the 6th day after inoculation. There was a significant increase in the number of cells in the spleen and bone marrow of animals treated with the extract in relation to the control, showing that these doses induce an increase in the production of peripheral immune cells and precursors.

#### 2.1.2. Glioblastoma

Glioblastomas are the most frequent and aggressive primary brain tumors, classified, according to the World Health Organization (WHO), as grade IV due to their malignancy [46]. Borges et al. [22] showed an in vitro antiproliferative effect of the ethanolic propolis extract produced by *Scaptotrigona* sp. against human adult glioblastoma cell lines (U251 and U343), with a decrease in cell proliferation by 48% to 59% in 72 h at a dose of 2 mg/mL, as well as a reduction in colony formation. They also observed that the combination of the propolis extract (2 mg/mL) with temozolomide (50 µM) had a synergistic antiproliferative effect, reducing cell proliferation to less than 20%. This association showed superior results related to the extract and drug when evaluated separately.

#### 2.1.3. Erythroleukemia

Erythroleukemia is a rare form of acute myeloid leukemia characterized by the proliferation of erythropoietic elements in the bone marrow; erythroblasts with foreign, lobulated nuclei; and pathological myeloblasts in the peripheral blood [47].

The mechanism of in vitro cytotoxicity of the ethanolic propolis extract produced by *Melipona orbignyi* against erythroleukemia cells (K562) was elucidated by Campos et al., [33], demonstrating that the cell viability decreased to less than 25% at the concentration of 500 μg/mL and that necrosis was the predominant form of cell death of cells treated with propolis. The data are interesting with regard to therapy against tumor cells resistant to cell death by apoptosis, which usually occurs with the use of conventional chemotherapy.

The in vitro cytotoxicity of the ethanolic propolis extract produced by *Tetragonisca fiebrigi* against erythroleukemia cells (K562) was higher with progressive increase in concentration. The most effective cytotoxic concentrations of the ethanolic extract propolis were 250 and 500 μg/mL, which promoted cell death by necrosis (23% ± 1.0% and 56% ± 1.4%) and secondary necrosis (10% ± 1.8% and 13% ± 0.8%), respectively. At the highest concentration evaluated (500 μg/mL), there was a 67% ± 2.5% reduction in viable cells [20].

Bonamigo et al. [31] observed the in vitro cytotoxic activity of ethanolic propolis extracts of *Scaptotrigona depilis* and *Melipona quadrifasciata anthidioides* against erythroleukemia cells (K562) as the concentration increased with cell growth of 32.6% ± 3.2% and 21.2% ± 4.1%, respectively. At the concentration of 500 μg/mL, by flow cytometry using the annexin and propidium iodide markers, after 24 h of treatment, the ethanolic propolis extract produced by *Scaptotrigona depilis* promoted death by necrosis in 52.9% ± 4.1% of the cells and death by late apoptosis in 12.1% ± 0.6% of the cells. The ethanolic propolis extract produced by *Melipona quadrifasciata anthidioides* promoted, after 24 h of treatment, death by necrosis in 57.5% ± 3.8% of the cells and death by late apoptosis in 19.4% ± 1.6% of the cells.

#### 2.1.4. Melanoma

Melanoma skin cancer originates from normal pigment cells called melanocytes. These melanocytes produce melanin, the pigment responsible for giving color to the skin and which protects the body from damage by the sun’s ultraviolet rays. Similar to other cells in the body, melanocytes can transform into cancer cells and when this transformation occurs, the result is the development of melanoma [48].

According to Cisilotto et al. [32], in vitro cytotoxicity of the hydroethanolic propolis extract produced by *Scaptotrigona bipunctata* against melanoma cells (SK-MEL-28) occurs by cell death by apoptosis, also evidenced in the accumulation of reactive oxygen species (ROS), reduction of mitochondrial membrane potential (Δψm), and induction of decreased levels of Bcl-2 proteins (antiapoptotic proteins) and AKT-3 (cell-growth-related protein). The extract also causes a decrease in migration and invasion of melanoma cells.

The combination of the extract (30 µg/mL) with the antineoplastic vemurafenib (15 μM) against melanoma cells demonstrated a synergistic effect, showing a cytotoxic effect, suggesting reduced resistance and increased cell death in cells with BRAF (proto-oncogene regulator of cell function) mutation.

#### 2.1.5. Osteosarcoma Cells

Osteosarcoma is a primary malignant bone tumor that can occur in any age group but mainly affects children, adolescents, and young adults and can also occur in animals [49]. Cinegaglia et al. [23] demonstrated that the hydroethanolic geopropolis extract produced by *Melipona fasciculata* exerts an in vitro cytotoxic effect against canine osteosarcoma cells in a dose- and time-dependent manner (24, 48, and 72 h). This was also evidenced by morphological analysis, showing the sensitivity of these cells to the extract.

#### 2.1.6. Laryngeal Carcinoma

Laryngeal carcinoma is among the most common head and neck cancers, accounting for about 2.4% of all newly diagnosed cases and 0.7% of all cancer-related deaths occurring worldwide/year [50].

Studies by Bartolomeu et al. [25] demonstrated in vitro cytotoxic activity of the hydroethanolic geopropolis extract produced by *Melipona fasciculata* against the growth of HEp-2 cells (larynx epidermoid carcinoma) and significant reduction in cell migration after 24 h of treatment with the extract. In the same study, the combination of the extract (25 mg/mL) with doxorubicin (1 mM) significantly affected the sensitivity of HEp-2 cells after 72 h, promoting apoptosis of this tumor lineage, presenting morphological changes, such as cytoplasmic membrane fragmentations (apoptotic bodies), loss of membrane, and integrity. Araújo et al. [30] also verified a significant decrease in cell viability observed after 6 h of incubation with 50 and 100 μg/mL of extract, and after 24, 48, and 72 h of incubation, there was a significant decrease in cell viability from 25 to 100 μg/mL.

#### 2.1.7. Ovarian Adenocarcinoma

Ovarian cancer is the second-most-common gynecological neoplasm, second only to cervical cancer. Almost all ovarian neoplasms (95%) are derived from the epithelial cells lining the ovary [51].

Da Cunha et al. [24] demonstrated the in vitro antiproliferative activity of the ethanolic geopropolis extract produced by *Melipona scutellaris* against ovarian adenocarcinoma with a multidrug-resistant phenotype (NCI/ADR-RES) and ovarian adenocarcinoma (OVCAR-03), with an IC_50_ range from 11.93 to 23.92 μg/mL. The total growth of these strains was inhibited at low concentrations when compared to normal strains (keratinocytes and normal murine fibroblast with IC_50_ of 43.20 and 52.73 μg/mL, respectively), thus demonstrating the selectivity of the ethanolic extract geopropolis for tumor cells.

The in vitro cytotoxicity of the hydroethanolic geopropolis extract produced by *Melipona fasciculata* (specifically at the concentration of 62.5 μg/mL, the highest exposed) against ovarian cancer lineage (A2780) was demonstrated through the visualization of several alterations in the morphology of these cells, such as cell rounding and shrinkage, presenting decreased density, standing out from the substrate. These characteristics are suggestive of cell death induced by the hydroethanolic extract mediated by apoptosis. This fact was confirmed with the increased expression of cleaved caspase-3 and of PARP, which is poly(ADP-ribose) polymerase cleaved by the Western blotting technique [8].

#### 2.1.8. Colorectal Adenocarcinoma

Colorectal cancer (CRC) accounts for 9% of all cancers worldwide, making it the second-most-common cancer in women and the third-most-common cancer in men. Adenocarcinoma arising from the rectal and colonic epithelium accounts for 90% of the CRC cases [52,53,54].

Umthong et al. [41] observed decreased cell viability for 23% of the colon cancer cells (SW620) treated with the aqueous propolis extract produced by *Trigona laeviceps*, and morphological changes were visualized in these cells, such as chromatin condensation, DNA fragmentation, internucleosomal DNA degradation, cell shrinkage, membrane blebbing, nuclear pyknosis, and apoptotic body formation, events typical of the apoptosis process.

Choudhari et al. [26] suggested that the hydroethanolic propolis extract produced by *Trigona* spp. exhibits in vitro cytotoxicity against human colon adenocarcinoma tumor cell lines (HT-29) as the incubation time and concentration were increased. The IC_50_ value found was 250 μg/mL. Morphological changes in cells demonstrated typical changes of apoptosis, i.e., apoptotic membrane blebbing and detachment of cells.

Kustiawan et al. [38] observed the in vitro cytotoxicity of methanolic, hexane, and ethyl acetate propolis extracts of *Trigona incisa*, *Trigona apicalis*, *Trigona fuscobalteata*, and *Trigona fuscibisca* species against human colon tumor cells (SW620), with IC_50_ ranging from 62 to 124 μg/mL.

#### 2.1.9. Carcinoma of the Pharynx

Pharynx cancer represents about 25% of malignant tumors affecting this area and 2% of all malignant diseases. The most prevalent histological type, in more than 90% of the patients, is squamous cell carcinoma [51].

Utispan et al. [36] evaluated the in vitro cytotoxicity of three fractions derived from the dichloromethane propolis extract produced by *Trigona sirindhornae* against cell lines derived from primary pharyngeal lesions (HN30) and lymph node metastases (HN31), both from the same patient. The fractions significantly decreased the viability of both cell lines at concentrations of 50 to 200 μg/mL.

#### 2.1.10. Gastric Adenocarcinoma

Stomach cancer is also called gastric cancer. The adenocarcinoma type accounts for about 95% of the stomach tumor cases and mostly affects men around 60–70 years of age. About 65% of the patients are over 50 years old [51].

Gastric cancer tumor lines (AGS, MKN-45, NUGC-4, and MKN-74) were treated in vitro with the ethanolic propolis extract produced by *Tetragonula biroi* by Desamero et al. [29], which revealed a proportional reduction in cancer cell proliferation as a function of higher concentration and longer incubation times, showing an IC_50_ range from 39 to 925 μg/mL after 72 h. Data confirmed in an in vivo assay using an animal model mimicking a gastric adenocarcinoma of a differentiated type indicated that after treatment with an ethanolic extract, there was a remarkable regression of macroscopic mucosal elevation, corresponding histologically to a substantial reduction in the pyloric mucosal thickness and infiltration of lymphocyte T.

In view of this, the evaluation of the antitumor activity of stingless bee products showed encouraging results. The predominance of in vitro studies to the detriment of animal assays was observed. In addition, the mechanism of cell death was not so explored and detailed in some studies. Sparse data from anticancer trials of stingless bees propolis and geopropolis demonstrate the need for these trials to prove the efficacy and safety of these products.

### 2.2. Chemical Identification of Antitumor Extracts from Propolis and Geopropolis

The chemical composition of propolis and geopropolis from stingless bees is shown in Table 2. The main classes of compounds identified are phenolics compounds (comprising phenolic acids, flavonoids, coumarins, and benzophenones) terpenes, steroids, alkaloids, fatty acids, and sugars. Qualitative approaches were used to define the classes of compounds; and analytical techniques, such as high-performance liquid chromatography coupled to mass spectrometry (HPLC/MS), liquid chromatography coupled to mass spectrometry (LC/MS), and gas chromatography coupled to spectrometry (GC/MS), were employed to identify the compounds.

β-amyrin, a compound identified in samples from stingless bees *Tetrigona apicalis*, *Scaptotrigona bipunctata*, *Melipona quadrifasciata anthidioides*, and *Melipona fasciculata*, was the subject of a study by Wen et al. [55] that highlighted the significant cytotoxic activity of this substance against HepG2 (hepatocellular carcinoma) cells. The cytotoxic effects were justified by the induction of apoptosis and the arrest of the G2/M cycle in a dose-dependent manner.

Cinnamic acid, a chemical compound identified in *Melipona orbignyi* and *Tetragonisca fiebrigi* samples, was explored by [56], who observed a reduced cell proliferation rate and a significant change in nuclear cytoplasmic ratio of nasopharyngeal carcinoma (NPC) after treatment with cinnamic acid. In addition, the treatment partially restored normal cell morphology and drove cell differentiation toward a benign phenotype and revealed cell death by apoptosis.

Ma et al. [57] showed that the compound taraxerone (identified in *Tetrigona apicalis* and *Melipona fasciculata* samples) exerts potent antiproliferative effects against A-549 (lung adenocarcinoma) in a strong dose-dependent and time-dependent manner. Furthermore, fluorescence microscopy revealed that taraxerone is able to induce cell shrinkage and chromatin condensation, recorded features of apoptosis.

p-Coumaric acid, identified in samples of species *Scaptotrigona bipunctata*, *Melipona quadrifasciata anthidioides*, and *Tetragonisca fiebrigi*, was studied by Sharma et al. [58], who observed significant inhibition of the proliferation of A375 (human melanoma) and B16 (mouse melanoma) cells after treatment with p-coumaric acid, as well as morphological changes in these cells after 48 h of treatment with different concentrations of the compound. They found increased levels of cleaved caspase-3 and cleaved caspase-9 in A375 and B16 cells, indicating that apoptosis is regulated by the family of caspases.

Artepillin C, present in the ethanolic extract produced by *Scaptotrigona bipunctata*, exhibited dose- and time-dependent cytotoxic effects on prostate cancer (HSC-3) cell lines. Flow cytometry analysis showed that 22% of the HSC-3 cells untreated with the compound suffered spontaneous cell death, while 77.32% of the cells were killed in response to the highest dose of artepillin C at 72 h. The antitumor activity of artepillin C is mediated by one of the following mechanisms: induction of cell cycle arrest in cancer cells, inhibition of angiogenesis, and inhibition of the oncogenic PAK1 signaling cascade [59].

Gallic acid, identified in the hydroethanolic extract geopropolis of *Melipona mondury* and in the ethanolic extract propolis of *Melipona quadrifasciata quadrifasciata*, inhibited the progression of prostate cancer cells (PC-3), was a mitochondrial potential enhancer (ΔΨm), and increased the number of apoptotic cells and DNA fragmentation. A Western blot analysis revealed negatively regulated expression of histone deacetylases (HDAC) 1 and 2, reported in various cancers, leading to the positive regulation of acetyl-p53 expression at the protein level, subsequent to the negative regulation of cell-cycle-related gene expression, i.e., proliferating cell nuclear antigen (PCNA) and cyclin D1 and E1; positively regulating the expression of the cell cycle arrest gene p21; and regulating the expression of genes related to the intrinsic apoptosis pathway, such as Bax, Bcl-2, cleaved caspase-3, and poly(ADP-ribose) polymerase [60].

Apigenin, presented in the ethanolic extract produced by *Melipona quadrifasciata anthidioides* propolis, inhibited proliferation, prevented cell cycle progression, and promoted apoptosis in both ovarian cancer cells (SKOV3) and cisplatin-resistant cells (SKOV3/DDP). In addition, apigenin reduced mitochondrial transmembrane potential and elevated caspase-3/cleaved caspase-3 and Bax/Bcl-2 ratios in both cell types. Quantitative reverse transcription PCR and Western blotting results demonstrated that apigenin significantly down-regulates Mcl-1 transcription and translation levels in SKOV3 and SKOV3/DDP cells, which is responsible for its cytotoxic functions and chemosensitizing effects [61].

Thus, the compounds identified in propolis and geopropolis extracts have already been studied by different researchers, demonstrating their antitumor potential. Phenolic compounds and terpenes were the most present classes of compounds in stingless bee products.

**Table 2:** Chemical composition of propolis and geopropolis extracts of stingless bee species.

Bee Species	Place of Origin	Product	Class of Compounds	Chemical Compounds	Method	Ref.
*Scaptotrigona bipunctata* (Latreille 1807)	Paraná/Santa Catarina, Brazil	Propolis	Alkaloids	Lelobanonoline, 2-[6-(2-hydroxy-propyl)-1-methyl-[2]piperidyl]-1-phenylethanone, norlobelanidine, norlobeline, lobeline, and lobelanidine	HPLC/MS	[17,32]
			Terpenes	α-Amyrin/β-amyrin and 4R,5R,9R,10R-13-hydroxypodocarp-8(14)-en-19-oic acid		
			Phenolic compounds (phenolic acids, flavonoids, coumarin, stilbenes, phenylpropanoids, and tannins)	Vicenin, liquiritigenin, formononetin, drupanin, p-coumaric acid, acid ferulic, biochanin A, kaempferol methyl ether, dihydrokaempferide, retusin 8-methyl ether, betuletol, artepillin C, 4-hydroxy-3(E)-(4-hydroxy-3- methyl-2-butenyl)-5-prenylcinnamic acid, 3-hydroxy-2,2-dimethyl-8-prenyl-2H-1-benzopyran-6-propenoic acid, artepillin C derivative, anacardic acid, dicaffeoylquinic, and (E)-3-{4-hydroxy-3-[(E)-4-(2,3-dihydrocinnamoyloxy)-3-methyl-2-butenyl]-5-prenylphenyl}-2-propenoic acid		
			Fatty acids	Palmitic acid, oleic acid, stearic acid, and eicosapentaenoic acid		
*Melipona fasciculata* (Smith 1854)	Maranhão, Brazil	Geopropolis	Phenolic compounds	Anacardic acid, heptedecenyl salicylic acid, nonadecenyl salicylic acid, pentadecenyl salicylic acid, heptadecadecylresorcinol, nonadecadecylresorcinol, pentadecadecadienylresorcinol, heptedecadienylresorcinol, taxifolin 7-*O*-rhamnoside, isoschaftoside, typhaneoside, dihydroquercetin-*C*-glycoside, narigenin-C-glycoside, vitexin-*O*-gallate, glycosylated pinobanksin, dihydroquercetin-3-O-rhamnoside, and gallocatechin-xylose	HPLC/MS	[8,30]
			Terpenes	Lupeol, α-amyrin, β-amyrin, α-amyrenone, β-amyrenone, triterpene ketone, taraxerone, dipterocarpol, marsformosanone, and 3-[Xyl]-28-Glc-phytophthalacagenin		
			Anthraquinone	Xantholaccaic acid A		
			Organic acids	Glycuronic acid, methylmalonic acid, and gluconic acid		
*Melipona scutellaris* (Latreille 1811)	Bahia, Brazil	Geopropolis	Benzofenones	Propensaeure 3-phenyl-trimethylsilylester and 1,2-benzenedicarboxylic acid	GC/MS	[24]
*Melipona quadrifasciata quadrifasciata* (Lepetetier 1836))	Paraná, Brazil	Propolis	Phenolic compounds	p-Coumaric acid, ferulic acid, ellagic acid, gallic acid, naringenin, aromadendrin, isosakuranetin, dihydrokaempferide, aromadendrin methyl ether, cinnamoyl-galloyl-hexoside, anacardic acid, cinnamoyl-coumaroyl-hexoside, dicoumaroyl-hexoside, digalloyl-cinnamoyl-hexoside, digalloyl-coumaroyl-hexoside, cinnamoyl-coumaroyl-galloyl hexoside, and dicoumaroyl-galloyl-hexoside	HPLC/MS	[17]
			Terpenes	Sugiol, pimaric acid, isocupressic acid, cupressic acid, junicedric acid, mangiferonic acid, and isomangiferolic acid		
*Melipona quadrifasciata anthidioides* (Lepeletier 1836)	Mato Grosso do Sul, Brazil	Propolis	Phenolic compounds	p-Coumaric acid, vanilic acid, caffeic acid, vanillin, ferulic acid, benzoic acid, quercetin, luteolin, cinnamic acid, and apigenin	HPLC/MS; GC/MS	[31]
			Terpenes	Stigmasterol, *β*-sitosterol, *β*-amyrin, taraxasterol, *α*-amyrin, *β*-amyrin acetate, and pinusenocarp		
*Melipona quadrifasciata anthidioides* (Lepeletier 1836)	Santa Catarina, Brazil	Propolis	Phenolic compounds	7-*O*-methyl aromadendrin, 5-hydroxy-4′,7-dimethoxy flavone, 2′-hydroxynaringenin, narigenin, and p-coumaric	HPLC/MS	[32]
			Phenylpropanoids	4-*O*-(6″-*O*-p-coumaroyl-β-D-glucopyranosyl)- and 6-*O*-cinnamoyl-1-*O*-*p*-coumaroyl-β-D-glucopyranoside		
			Terpenes	Abieta-8,11,13,15-tetraen-18-oic acid, abietic acid, 7-hydroxydehydroabietic acid, and inumakiol		
*Melipona orbignyi* (Guérin-Méneville 1844)	Mato Grosso do Sul, Brazil	Propolis	Phenolic compounds	Dihydrocinnamic acids, cinnamic acids, benzoic acids, coumarin C-prenylated acids, and long-chain caffeates	GC/MS	[33]
*Melipona mondury* (Smith 1863)	Bahia, Brazil	Geopropolis	Phenolic compounds	Gallic acid	HPLC/MS	[15]
			Terpenes	Not specified		
*Trigona* spp.	Maharashtra, India	Propolis	Unidentified	Unidentified	-	[26]
	Indonesia	Propolis	Alkaloids, flavonoids, saponins, tannins, steroids, and triterpenes	Unidentified	Chemical approach	[34]
*Scaptotrigonaaff. postica* (Latreille 1807)	Maranhão, Brazil	Propolis	Terpenes and coumarins	Unidentified	Phytochemical approach	[28]
*Scaptotrigona depilis* (Moure 1942)	Mato Grosso do Sul, Brazil	Propolis	Terpenes	*β*-Sitosterol, *β*-amyrin, *α*-amyrin, and *β*-amyrin acetate	GC/MS; HPLC/MS	[31]
			Phenolic compounds	Vanillin, p-coumaric acid, ferulic acid, benzoic acid, and cinnamic acid		
*Tetragonula biroi* (Friese 1898)	Lagunas, Philippines	Propolis	Carbohydrates, steroids, alkaloids, anthraquinones, and phenols	Unidentified	Phytochemical approach	[29]
*Tetragonisca fiebrigi* (Schwartz 1938)	Mato Grosso do Sul, Brazil	Propolis	Phenolic acids	Benzoic acid, cinnamic acid, p-coumaric acid, 3-phenyl-p-coumaric acid, and benzyl caffeate	GC/MS	[20]
			Phenylpropanoids	Cinnamyl caffeate, hydrocinnamic acid, and hydrocinnamic acid ethyl ester		
			Terpene	Kaurenoic acid		
			Sugars	Fructose and glucose		
			Lipids	Tocopherol, cholesterol, and retinol		
*Tetrigona apicalis* (Smith 1857)	Perak, Malaysia	Propolis	Hydrocarbon	Undecane	GC/MS	[44]
			Phenolic compound	Myristicin		
			Terpenes	β-Elemene, α-cubebene, copaene, cyperene, α-gurjunene, caryophyllene, α-caryophyllene, γ -cadinene, germacrene D, bicyclogermacrene, δ-amorphene, β-selinene, aromadendr-1-ene, spathulenol, caryophyllene oxide, 1, 2-dimethyl-3, 5-bis(1-methylethenyl)-, humulene epoxide II, α-cadinol, aristolene epoxide, taraxerone, β-amyrin, and α-amyrin		
*Plebeia remota* (Holmberg 1903)	Paraná, Brazil	Propolis	Fatty acid	Arachidonic acid	HPLC/MS	[17]
			Terpenes	Sugiol, totarol, communic acid, agathic acid, isocupressic acid, cupressic acid, dihydroagathic acid, and 15-acetoxy-cupressic acid		

HPLC/MS = high-performance liquid chromatography coupled to mass spectrometry. GC/MS = gas chromatography coupled to mass spectrometry.

### 2.3. Isolation of Compounds

Compounds isolated from propolis and geopropolis of stingless bee species that were tested against tumor cell lines are shown in Figure 2.

**Figure 2:**
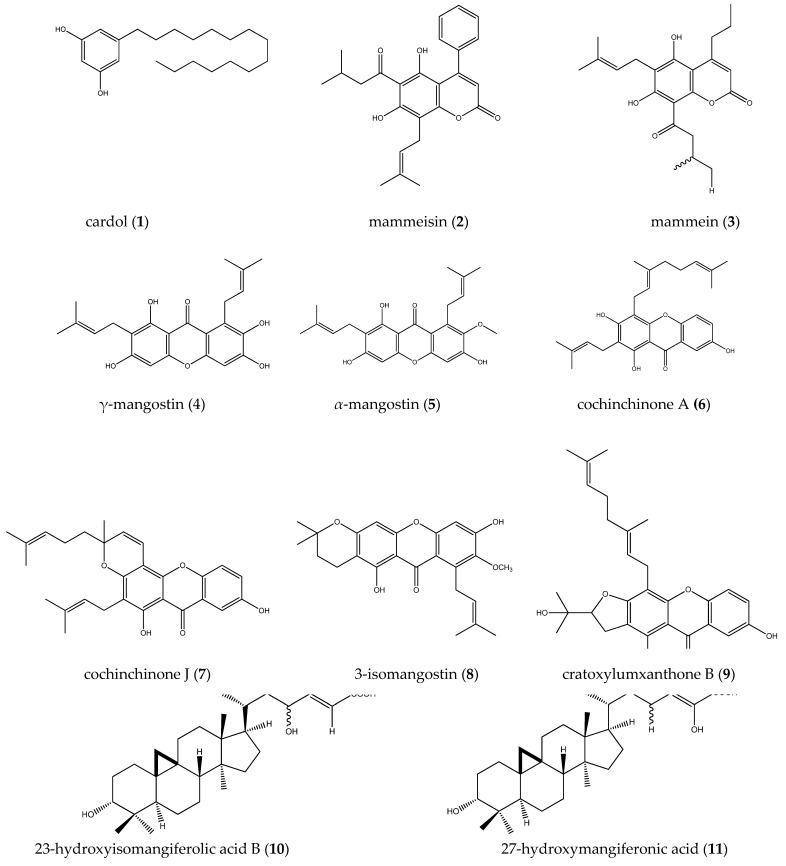

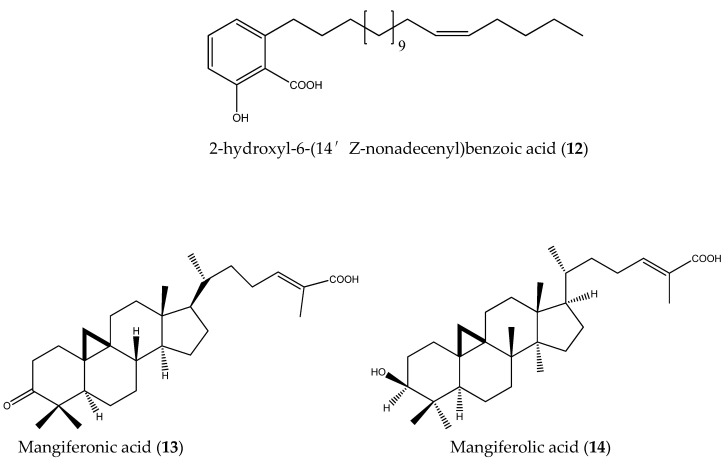
Compound **1** was isolated from the methanolic extract propolis of *Trigona incisa*. Compounds **2** and **3** were isolated from the hydroethanolic extract geopropolis of *Melipona scutellaris.* Compounds **4** and **5** were isolated from the methanolic extract propolis of *Tetragonula pagdeni.* Compounds **4**, **6**, **7**, **8**, and **9** were isolated from the hydroethanolic extract propolis of *Lisotrigona furva*. Compounds **10**, **11**, and **12** were isolated from the ethanolic extract propolis of *Trigona* minor. Compound **13** was isolated from the ethanolic extract propolis of *Homotrigona fimbriata*. Compound **14** was isolated from the acetate extract propolis of *Lisotrigona furva*.

Kustiawan et al. [38,39,40] submitted the methanolic propolis extract produced by *Trigona incisa* to chromatographic fractionation, isolating, among other compounds, cardol (**1**), which was identified by NMR spectrometric analysis. Biological cytotoxicity tests indicated that compound **1** induces cell death by apoptosis in the initial incubation period (≤6 h) and modulates cell cycle arrest in the G1 subphase in SW620 cells (colon cancer cells). Kustiawan et al. [40] observed that compound **1** promotes changes in cell morphology in SW620 cells; a significant increase in caspase-3 and caspase-9 activities; and cleavage of pro-caspase-3, pro-caspase-9, and PARP.

Eight compounds were isolated from the hydroethanolic geopropolis extract produced by *Melipona scutellaris* by [62] and tested in vitro against two colon cancer cell lines (COLO205 and KM12). The coumarins mammeisin (**2**) and mammein (**3**) (Figure 2) showed a higher average percentage of growth inhibition, of 56% and 83%, respectively. The mechanism of cytotoxicity of the extract against tumor cells was not investigated by the authors.

Vongsak et al. [27] tested the in vitro cytotoxicity of the methanolic propolis extract produced by *Tetragonula pagdeni* against oral squamous cell carcinoma (KB), hepatocellular carcinoma (HepG2), colon adenocarcinoma (CaCo-2), and melanoma (SK-MEL-28) cell lines and observed variation in IC_50_ values from 33. 38 to 80.81 μg/mL. In contrast, the IC_50_ value of normal human fibroblast cells was 228.75 μg/mL, demonstrating greater selectivity of propolis metabolites toward tumor cells. From the said extract, γ-mangostin (**4**) and α-mangostin (**5**) were isolated by preparative thin layer chromatography and their structures identified by spectrometric methods (NMR^1^H and ^13^C) (Figure 2). These substances expressed comparable cytotoxicity to the positive control, doxorubicin, against these tumor cell lines, with IC_50_ values from 2.84 to 15.12 μg/mL and 1.63 to 7.07 μg/mL for (**4**) and (**5**), respectively.

Twenty-three chemical compounds were isolated from the hydroethanolic propolis extract produced by *Lisotrigona furva* by Oanh et al. [63], among which, cochinchinone A (**6**), cochinchinone J (**7**), cratoxylumxanthone B (**8**), 3-isomangostin (**9**), and γ-mangostin (**4**)**,** (Figure 2) were tested against mouth epidermoid carcinoma (KB), human hepatoma (HepG-2), human lung adenocarcinoma (SK-LU-1), and human breast adenocarcinoma (MCF-7). These compounds showed activity on the tested tumor cell lines, with compound (**4**) demonstrating greater activity compared to others, showing an IC_50_ value of 2.10 and 2.73 μg/mL, respectively, in human hepatoma and human lung adenocarcinoma.

The ethanolic propolis extract produced by *Trigona minor* was subjected to partionation with solvents of different polarities. The n-hexane extract showed the most potent preferential cytotoxicity against human pancreatic cancer cells (PANC-1), with an IC_50_ value of 3.6 μg/mL. Further separation and purification of this fraction led to the identification of 16 triterpenoids, most notably 23-hydroxyisomangiferolic acid B (**10**) and 27-hydroxyisomangiferolic acid (**11**), which showed stronger preferential cytotoxicity, with IC_50_ values of 4.3 and 3.7 μM, respectively. Subsequently, compound (**10**) was evaluated for its effect on the cell morphology of PANC-1 cells. When these cells were treated with 5 μM of compound (**10**) for 24 h, the PANC-1 cells changed morphologically and gave a unique red fluorescence, indicating the apoptosis process. Furthermore, in the colony formation assay in PANC-1 cells, compound (**10**) significantly inhibited colony formation in a concentration-dependent manner [64].

Three more substances were isolated from the ethanolic propolis extract produced by *Trigona minor*, with emphasis on 2 hydroxyl-6- (14′Z-nonadecenyl) benzoic acid (**12**), which showed preferential cytotoxicity against the human pancreatic cell line PANC-1, with an IC_50_ value of 2.4 μM. The cytotoxicity of this compound is related to the substituents on the alkenylphenol ring. The presence of the carboxylic acid group or one more hydroxyl group appears to increase the activity [65].

The ethanolic propolis extract produced by *Homotrigona fimbriata* was fractionated by silica gel column chromatography, leading to the isolation of mangiferonic acid (**13**), which showed moderate cytotoxicity, with IC_50_ = 96.76 mM in MCF-7 cells, IC_50_ > 110.04 mM in HeLa cells, and IC_50_ > 110.04 mM in CaCo-2 cells [37].

Chromatographic separation of the ethyl acetate propolis extract produced by *Lisotrigona furva* led to the isolation of five cycloartane-type triterpenes, which were tested on lung cancer cell lines (LU-1) and breast cancer cell lines (MCF-7), most notably mangiferolic acid (**14**), which showed an IC_50_ value of 13.33 and 62.85 μg/mL, respectively [66].

## 3. Materials and Methods

This review covered ScienceDirect, Scopus, Pubmed, and Scielo databases, as updated on October 2021. The references obtained in the review were consulted and analyzed in detail. The key words employed alone or in combination in the literature review were propolis, geopropolis, stingless bee, cancer, cytotoxicity, and antiproliferative. Articles on propolis from sting bee species (i.e., *Apis mellifera*) were excluded from the search.

## 4. Conclusions

The propolis and geopropolis extracts from stingless bees analyzed in this revision had diverse and complex chemical compositions, and their constituents belong to the chemical classes of phenolic acids, flavonoids, coumarins, benzophenones, terpenes, steroids, alkaloids, fatty acids, and sugars. The extracts and isolated substances showed selective cytotoxicity against different tumor cell lines, suggesting an antineoplastic potential and synergism with standard chemotherapeutics. Although the preliminary results of propolis and geopropolis from stingless bees are encouraging, further preclinical studies and clinical trials are essential to validate the safety, efficacy, and effectiveness of the products from these species in cancer therapy.

## Data Availability

Data sharing not applicable.
